# Population‐specific single‐nucleotide polymorphism confers increased risk of venous thromboembolism in African Americans

**DOI:** 10.1002/mgg3.226

**Published:** 2016-06-21

**Authors:** Roxana Daneshjou, Larisa H. Cavallari, Peter E. Weeke, Konrad J. Karczewski, Katarzyna Drozda, Minoli A. Perera, Julie A. Johnson, Teri E. Klein, Carlos D. Bustamante, Dan M. Roden, Christian Shaffer, Joshua C. Denny, James L. Zehnder, Russ B. Altman

**Affiliations:** ^1^ Department of GeneticsStanford University School of MedicineStanfordCalifornia94305; ^2^ Department of Pharmacotherapy and Translational ResearchUniversity of FloridaGainesvilleFlorida32610; ^3^ Department of MedicineVanderbilt UniversityNashvilleTennessee37201; ^4^ The Department of CardiologyCopenhagen University HospitalGentofteDenmark; ^5^ Biomedical Informatics Training ProgramStanford University School of MedicineStanfordCalifornia 94305; ^6^ Department of Pharmacy PracticeUniversity of Illinois at ChicagoChicagoIllinois60612; ^7^ Department of MedicineUniversity of ChicagoChicagoIllinois60637; ^8^ Department of PharmacologyVanderbilt UniversityNashvilleTennessee37201; ^9^ Department of BiomedicalInformatics Vanderbilt UniversityNashvilleTennessee37201; ^10^ Department of MedicineStanford University School of MedicineStanfordCalifornia94305; ^11^ Department of BioengineeringStanford University School of MedicineStanfordCalifornia94305

**Keywords:** African American, hypercoagulability, pulmonary embolism, venous thromboembolism

## Abstract

**Introduction:**

African Americans have a higher incidence of venous thromboembolism (VTE) than European descent individuals. However, the typical genetic risk factors in populations of European descent are nearly absent in African Americans, and population‐specific genetic factors influencing the higher VTE rate are not well characterized.

**Methods:**

We performed a candidate gene analysis on an exome‐sequenced African American family with recurrent VTE and identified a variant in Protein S (*PROS1*) V510M (rs138925964). We assessed the population impact of *PROS1* V510M using a multicenter African American cohort of 306 cases with VTE compared to 370 controls. Additionally, we compared our case cohort to a background population cohort of 2203 African Americans in the NHLBI GO Exome Sequencing Project (ESP).

**Results:**

In the African American family with recurrent VTE, we found prior laboratories for our cases indicating low free Protein S levels, providing functional support for *PROS1* V510M as the causative mutation. Additionally, this variant was significantly enriched in the VTE cases of our multicenter case–control study (Fisher's Exact Test, *P* = 0.0041, OR = 4.62, 95% CI: 1.51–15.20; allele frequencies – cases: 2.45%, controls: 0.54%). Similarly, *PROS1* V510M was also enriched in our VTE case cohort compared to African Americans in the ESP cohort (Fisher's Exact Test, *P* = 0.010, OR = 2.28, 95% CI: 1.26–4.10).

**Conclusions:**

We found a variant, *PROS1* V510M, in an African American family with VTE and clinical laboratory abnormalities in Protein S. Additionally, we found that this variant conferred increased risk of VTE in a case–control study of African Americans. In the ESP cohort, the variant is nearly absent in ESP European descent subjects (*n* = 3, allele frequency: 0.03%). Additionally, in 1000 Genomes Phase 3 data, the variant only appears in African descent populations. Thus, *PROS1* V510M is a population‐specific genetic risk factor for VTE in African Americans.

## Introduction

Venous thromboembolism (VTE), consisting of deep vein thrombosis (DVT), pulmonary embolism (PE) or both, has an annual incidence of 300,000 to 900,000 cases in the United States alone and is a cause of significant mortality and morbidity (Beckman et al. 2010; Raskob et al. 2010). African Americans have a 30–60% higher incidence of VTE than individuals of European descent (Roberts et al. 2009; Zakai and McClure 2011).

The risk factors for VTE are complex and include environmental risk factors (e.g., vessel injury and blood stasis) and genetic risk factors including common and/or rare variants that predispose to hypercoagulation (Feero 2004). Genetic risk factors for thrombophilia have been extensively studied in European descent populations (Gandrille et al. 2000; Gohil et al. 2009; Rosendaal and Reitsma 2009). Clinically tested genetic variants in European descent individuals include *F5* R506Q (5% prevalence) (MIM#: 612309), which confers a three to fivefold increased risk of VTE in carriers and *F2* G20210A (0.7 to 4% prevalence) (MIM#: 176930), which confers a two to threefold increased risk of VTE in carriers (Middeldorp and van Hylckama Vlieg 2008; Rosendaal and Reitsma 2009). However, both of these variants are rare in individuals of African descent (Dowling et al. 2003; Roberts et al. 2009). A review of the literature shows a dearth of identified genetic risk factors for VTE in African Americans, even though African Americans have a similar rate of positive family history as European descent individuals (28–29%) (Dowling et al. 2003).

Vitamin K‐dependent Protein S (*PROS1*, MIM#: 176880) is a cofactor of the anticoagulant enzyme activated protein C (APC), a protease which cleaves procoagulant Factors Va and VIIIa (Garcia de Frutos and Fuentes‐Prior 2007). Damaging mutations in Protein S cause hereditary VTE in populations of European descent, with a fivefold higher relative risk in familial carriers (Gandrille et al. 2000; Makris et al. 2000; Duebgen et al. 2012; Holzhauer et al. 2012). Recent research recommends genetic screening in cases of hereditary thrombophilia caused by *PROTEIN C* (MIM#: 612283), *PROTEIN S*, or *ANTITHROMBIN III* (MIM#: 107300) mutations because of a significantly increased risk of VTE in carriers versus noncarriers (Holzhauer et al. 2012). We present a previously uncharacterized Protein S mutation specific to African Americans that associates with VTE in this understudied cohort.

## Methods

### Ethical compliance

All patients provided written informed consent for study participation according to an institutional review board‐approved protocol at each participating site. BioVU, the Vanderbilt DNA biobank, accrued subjects using an opt‐out approach as previously described (Roden et al. 2008).

### Family with venous thromboembolism

We identified an African American family (mother and two adult daughters) with recurrent deep vein thrombosis (DVT)/pulmonary embolism (PE) during a warfarin pharmacogenetic exome sequencing study. An initial analysis failed to identify *F5* R506Q or *F2* G20210A, European variants that cause hereditary VTE.

Genetic information from unaffected family members was not available. Depicted in Figure S1 is the pipeline we developed to analyze exome data in the three affected family members. First, we filtered for heterozygous variants shared among the family members. Next, we filtered by a list of candidate genes – *F5* (NCBI RefSeq NG_011806.1), *PROTEIN C* (NCBI RefSeq NG_016323.1), *PROTEIN S* (NCBI RefSeq NG_009813.1), *PROTHROMBIN* (NCBI RefSeq NG_008953.1), *ANTITHROMBIN III* (NCBI RefSeq NG_012462.1), *CYSTATHIONINE BETA‐SYNTHASE* (NCBI RefSeq NG_008938.1), *C4BPA* (NCBI RefSeq NG_029386.1), *C4BPB* (NCBI RefSeq NG_029386.1) – previously implicated in VTE in other populations (Feero 2004; Martinelli et al. 2004; Buil et al. 2010). Within these genes, we filtered for amino acid changing mutations (nonsynonymous) with a population minor allele frequency less than 2% among African Americans in the Exome Variant Server (EVS), created from the NHLBI GO Exome Sequencing Project (ESP) (NHLBI GO Exome Sequencing Project [ESP]). A population minor allele frequency <1% is considered a rare variant; we filtered at double that threshold in order to capture any borderline rare variants (Li et al. 2013). Mutational effects were evaluated using SIFT and Polyphen (Kumar et al. 2009; Adzhubei et al. 2010). Finally, we extracted hematological laboratory results from the electronic medical system for the three family members. Laboratory results were generated by the the academic medical center's clinical pathology laboratory, and Protein S assays were done using an Immunoturbidimetric‐latex based method, with a reference range of 60% to 140%.

### Population‐level data

Population level data on variant frequencies were obtained using data from the ESP, the 1000 Genomes Phase 3, and the ExAC Browser (NHLBI GO Exome Sequencing Project (ESP); Lek et al. 2015).

### Case–control analysis of candidate variant

To assess the population impact of the variant we identified, we performed a case–control analysis using a multicenter cohort with a total of 306 cases and 370 controls. The cohort is made of two subcohorts.

### Warfarin subcohort

The warfarin subcohort consisted of 102 African American individuals from the University of Chicago, the University of Illinois, and the University of Florida, who were previously studied by exome sequencing for a warfarin pharmacogenomics study (Daneshjou et al. 2014). Since VTE status is an important covariate for predicting warfarin dose, any history of VTE was documented on all of these individuals: VTE status was identified from the electronic medical record (EMR) as documented based on Doppler/Duplex ultrasound results for DVT and Ventilation/Perfusion scan for PE. A total of 65 of these individuals had documented pulmonary embolism or deep vein thrombosis. In many cases, VTE was the indication for being placed on anticoagulation. The remaining 37 individuals were on warfarin for other reasons (heart valve replacement, atrial fibrillation, stroke, peripheral vascular disease) and did not have a history of VTE. These individuals were used as controls.

In addition, we genotyped our variant of interest in an 108 cases and 97 controls from the University of Florida and the University of Illinois who met the same criteria as the exome sequenced cohort – on warfarin with VTE and on warfarin without VTE, respectively (Perera et al. 2013). VTE status was determined as described above. We did not have information on age at VTE event; however, we did know age at the time of enrollment into the warfarin study. Other available covariates included sex, weight at enrollment, and height at enrollment (Table S2). For the warfarin subcohort, African ancestry was confirmed using ancestry informative markers, as previously described (Falush et al. 2003; Robbins et al. 2007). The total number of subjects from this subcohort was 173 cases and 134 controls.

Additionally, we used the exome sequenced warfarin cohort to assess whether our variant of interest was in linkage disequilibrium (LD) with any neighboring variants using the R2 command in PLINK v1.90b3s (Purcell S. et al. 2007; Daneshjou et al. 2014; Chang et al. 2015).

### Vanderbilt BioVU subcohort

We used Vanderbilt BioVU, a database with deidentified EMR tied to DNA samples (Roden et al. 2008; Ritchie et al. 2010), to identify additional cases and controls, who were then genotyped at *PROS1* V512M using a Taqman SNP genotyping assay. We selected individuals of self‐reported African descent and used ICD9 codes for DVT or PE to identify cases and controls. For cases, we selected individuals who were less than 50 years old with an event, and for controls, we selected individuals who were greater than 70 years old and did not have an event. Additional available covariates included sex, weight, and height (Table S2). Previously, it has been shown that the self‐reported African descent in the Vanderbilt BioVU Cohort highly correlates with actual African ancestry (Dumitrescu et al. 2010). Of samples from 137 cases and 239 controls sent for genotyping, genotyping was successful on 133 cases and 236 controls.

### NHLBI GO Exome Sequencing Project data

The NHLBI GO ESP includes exome sequence data on 2203 African Americans; genotype distributions from this dataset were used as an additional larger control cohort (NHLBI GO Exome Sequencing Project [ESP]). We compared the distribution of the variant of interest in our Warfarin cohort and Vanderbilt BioVU cases to this large background cohort.

### Sequencing and genotyping

Exome sequencing procedures were done as previously described (Daneshjou et al. 2014). Genotyping of the warfarin genotyped subcohort was done using pyrosequencing; information about the primers can be found in the Figure S2. Genotyping for the Vanderbilt BioVU cohort was done using a Custom TaqMan SNP genotyping assay according to the manufacturers’ recommended protocols. Probes and primers were designed and synthesized by Life Technologies (Carlsbad, CA). Genotype calls were determined by individuals not familiar with case/control status. Information about the primers can be found in Figure S3. Quality control measures for subcohorts can be found in Exhibit S1.

### Statistical analysis

We compared the distribution of the risk alleles between cases and controls using the Fisher's Exact Test. We assessed male versus female distribution of the risk variant in the cases using a binomial test. Comparisons on covariates between cases and controls in each subcohort were done using the *t*‐test for continuous variables and Fischer's exact test for categorical variables. We demonstrated the covariates that were significantly different between cases and controls were not confounding our risk variant using logistic regression. All statistical analysis was done in the statistical programming package R (v. 2.15.3); Fisher's Exact Test was done using the exactci_1.2‐0 library in R (Fay 2010).

## Results

### Protein S nonsynonymous mutation in African American family with hereditary venous thromboembolism

**Figure 1:**
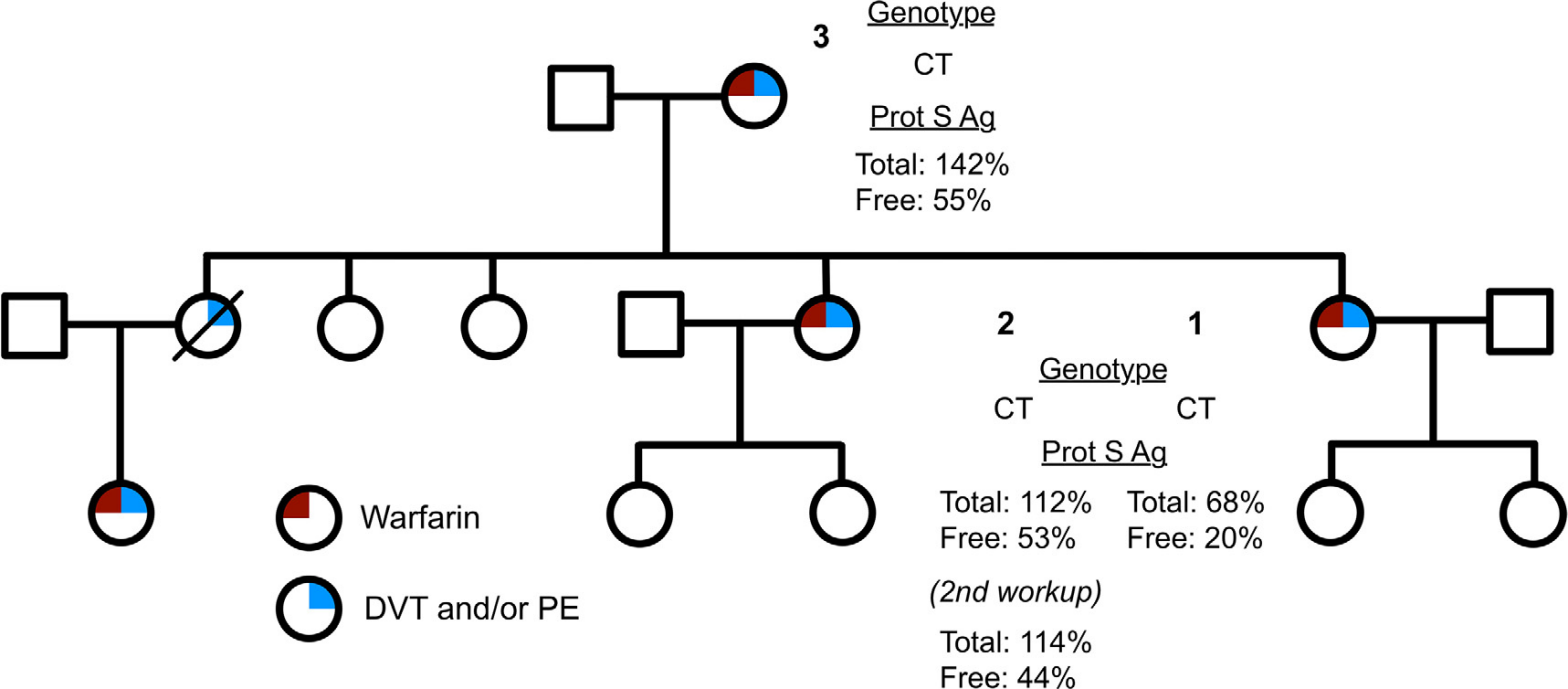
Pedigree of the family with hereditary venous thromboembolism. *PROS1* Val510Met genotype and Protein S antigen labs (reference range 60% to 140%) are listed.

Using family history as reported by index subject 1, we constructed a pedigree of the family, showing the history of VTE and use of warfarin as treatment (Fig. 1). We had exome sequence data from three of these family members – subjects 3, 2, and 1, a mother and two adult daughters. Using these exome data, we filtered for heterozygous, shared, deleterious variation at low frequency in genes previously implicated in clotting. A single variant, rs138925964, passed all filters. This variant changes a valine to methionine at position 510 of vitamin K‐dependent Protein S (*PROS1*) and is predicted to be “damaging” by SIFT and “possibly damaging” by Polyphen (Kumar et al. 2009; Adzhubei et al. 2010).

We evaluated this candidate variant clinically by mining the clinical data of available family members for supporting laboratory information. Clinical workup indicated low free Protein S in all three family members during periods when warfarin was not being prescribed, and two out of three had a documented history of Protein S deficiency (Fig. 1). Subject 1 also had an autoimmune disorder, but laboratory results did not indicate antiphospholipid syndrome. A full description of the past medical history and normal laboratory values for the three family members is presented in Table S1.

### *PROS1* V510M specific to African descent populations

*PROS1* V510M has a minor allele frequency (MAF) of 0.03% (3/4300 subjects are heterozygote carriers) in European descent populations and 1.09% (48/2203 subjects are heterozygote carriers) in African American descent populations in the EVS (NHLBI GO Exome Sequencing Project [ESP]). In 1000 Genomes Phase 3 data, the variant is only seen in populations of African descent – African Caribbeans in Barbados (MAF = 1.42%, *n* = 96), Gambian in the Western Divisions in The Gambia (MAF = 0.88%, *n* = 113), Esan in Nigeria (MAF = 0.51%, *n* = 99), Yoruba in Ibadan, Nigeria (MAF = 0.46%, *n* = 109), Luhya in Webuye, Kenya (0.50%, *n* = 101), and African ancestry in Southwest United States (0.76%, *n* = 66). In the ExAC aggregation consortium, which includes the EVS dataset, 1000 genomes, as well as several other African American and African descent populations, the variant frequency is 1.096% for African descent populations (*n* = 5202) (Exome Aggregation Consortium (ExAC); Lek et al. 2015).

### *PROS1* V510M associated with increased VTE risk in African Americans

We found the risk allele to be greatly enriched in the 306 subjects with VTE (MAF = 2.45%) compared to the 370 controls (MAF = 0.54%) (*P* = 0.0041, OR = 4.62, CI = 1.51–15.20). The variant was statistically enriched in the VTE group (*P* = 0.010, OR = 2.28, CI = 1.26–4.10) compared to the 2203 African Americans in the EVS.

Furthermore, *PROS1* V510M was not found to be in LD with any other variants in the warfarin subcohort exome data.

The additional clinical covariates for the cohort can be found in Table S2. We found no differences in sex between cases and controls. Furthermore, even though all the cases in the index family were women, there was not a statistical enrichment of women carrying the variant in the population analysis. There was a statistically significant difference in age at enrollment between cases and controls; however, in the Vanderbilt BioVU subcohort, this difference was created by the selection criteria. There were differences in weight and height between cases and controls; however, a logistic regression to model case/control status of the cohort using height, weight and the risk variant showed that the risk variant was still a predictor for VTE status, even after correcting for height and weight differences (Table S3).

## Discussion

We present a population‐specific, previously uncharacterized nonsynonymous mutation in Protein S, V510M, which associates with VTE in African Americans, a population whose genetic risk factors for VTE have been poorly characterized (Dowling et al. 2003).

Protein S plays an important role in anticoagulation as a cofactor of the anticoagulant enzyme activated protein C (APC) and is also reported to have its own independent anticoagulant activity (Heeb et al. 1993; Garcia de Frutos and Fuentes‐Prior 2007). In European populations, damaging mutations in Protein S are rare and have primarily been described in families with hereditary VTE (Gandrille et al. 2000; Rosendaal and Reitsma 2009). In fact, in a European family, an amino acid change at the position neighboring our described variant, *PROS1* Leu511Ser, results in Protein S deficiency and thrombophilia (Mustafa et al. 1995). However, there is also evidence of population‐level mutations in PROS1 affecting disease risk: in the Japanese population, the Protein S Tokushima K196E is associated with increased VTE risk and is estimated to have a population prevalence of 0.9% to 1.6% (Kimura et al. 2006; ten Kate and van der Meer 2008). Our variant, *PROS1* V510M, has a similar population prevalence from 0.5% to 1.42% among African Americans and African descent populations in the EVS, 1000 Genomes Phase 3, and ExAC, and is virtually absent in other populations.

African Americans have a higher rate of VTE than European descent populations, but the genetic factors commonly tested in European descent populations are far less prevalent in African populations (Roberts et al. 2009). The genetic risk factors for VTE specific to African Americans have not been well characterized. Since VTE genetic risk factors tend to be at lower frequencies in the population, genome‐wide association studies are often underpowered to discover them. However, here, we were able to identify *PROS1* V510M using a family with hereditary VTE whose functional workup supported our identified variant: all family members had low free Protein S. As has been shown in previously family studies, genetic mutations in Protein S associated with low free Protein S levels increase the risk of a thrombotic event (Gandrille et al. 2000). *PROS1* V510M is in the sex hormone binding globulin domain (named for its similarity to sex hormone binding globulins, but not thought to actually bind sex hormones). This region has been experimentally shown to be involved in optimal APC cofactor activity and in Protein S's binding to the plasma protein C4BP, which determines the availability of free Protein S (van Wijnen et al. 1998).

Our study had some limitations. African Americans with well‐documented DVT/PE phenotypes and available DNA samples were limited, which affected our sample size; however, despite being underpowered, we detected a statistically significant signal. This signal is unlikely to be driven by any particular subcohort since the allele frequencies of *PROS1* V510M among the cases and controls in each subcohort were not statistically different (Exhibit S1). Moreover, our sample size was similar to previously published studies in African Americans, a population which is underrepresented in genetic studies (Daneshjou et al. 2014). In order to have a comparison of our cases to the background African American population, we used data from 2203 individuals in EVS. This allowed us to demonstrate that our association is robust to using a larger population background as the control group. However, the EVS individuals did not have any information on DVT/PE phenotypes, and thus, the analysis between our cases and that cohort may actually underpredict the effect of the mutation.

Additionally, there are differences in the clinical features of our subcohorts. All the individuals in the Warfarin subcohort were on the anticoagulant warfarin, which is used to treat VTE for at least 3 months (Baglin et al. 2012). The controls from this cohort were on warfarin for non‐VTE reasons; however, these individuals would have been subsequently protected from VTE due to the anticoagulation. The Vanderbilt BioVU subcohort was selected using age constraints to ensure controls were older and unlikely to have an event subsequently. Because the cases were younger than 50, they were more likely to be individuals who have a genetic predisposition to VTE rather than due to secondary nongenetic causes such as malignancy or immobilization. Information about whether our cases had provoked or unprovoked VTE was unavailable. Since VTE is a multifactorial disease often with some combination of stasis, endothelial injury and hypercoagulability, even individuals with genetic risk factors will often have additional precipitating factors (Feero 2004). Additionally, while the index family had information on Protein S laboratory data, this information was unavailable or not noted on our population study. However, previous studies in European families with protein S mutations have shown inconsistency between genotype and Protein S laboratory findings; the presence of deep vein thrombosis serves as a more reliable and clinically relevant phenotype (ten Kate and van der Meer 2008).

In our study, we found differences in weight and height between the cases and controls. A study had previously found that obesity and tall stature were associated with VTE risk; however, in our population, obesity and short stature were associated with VTE risk (Borch et al. 2011). However, most importantly, we showed that even in accounting for these differences in the case–control cohort, the genetic risk factor is still a statistically significant predictor of VTE.

Given the prevalence of VTE, the odds ratio approximates to the relative risk, meaning that our variant confers 2.3–4.6× increased risk of VTE in the African American population. This increase in risk is consistent with the increased risk seen in commonly clinically tested genetic risk factors in European descent populations, such as *F5* R506Q or *F2* G20210A (Middeldorp and van Hylckama Vlieg 2008; Rosendaal and Reitsma 2009). We know from studying other disease processes that population‐specific genetic variation plays an important role in disease risk (Rosenberg et al. 2010). Therefore, discovering genetic risk factors for thrombophilia in African Americans, such as *PROS1* V510M, will be instrumental to implementing inclusive precision medicine.

## Conflict of Interest

RBA is founder and consultant to Personalis. CDB is on the advisory board of a project at 23andMe; and on the scientific advisory boards of Personalis, Inc.; InVitae; Etalon, Inc.; and Ancestry.com. TEK is a consultant to Personalis.

## Supporting information


**Figure S1.** Analysis process for identifying variants of interest in an African American family with a history of venous thromboembolism.
**Figure S2.** Description of Primers used for Pyrosequencing of SNP.
**Figure S3.** Description of Primers used for Taqman SNP genotyping assay.
**Table S1.** Clinical data of the African American family with hereditary VTE.
**Table S2.** Covariates for Warfarin and Vanderbilt subcohorts. *Denotes statistical difference between cases and controls in a subcohort.
**Table S3.** Logistic model for population cohort using height, weight, and risk variant. 98 samples were excluded due to missingness in height or weight.
**Exhibit S1.** Quality Control Measures.

## References

[mgg3226-bib-0001] Adzhubei, I. A. , S. Schmidt , L. Peshkin , V. E. Ramensky , A. Gerasimova , P. Bork , et al. 2010 A method and server for predicting damaging missense mutations. Nat. Methods 7:248–249. 20354512 10.1038/nmeth0410-248 PMC2855889

[mgg3226-bib-0002] Baglin, T. , K. Bauer , J. Douketis , H. Buller , A. Srivastava , and G. Johnson . 2012 Duration of anticoagulant therapy after a first episode of an unprovoked pulmonary embolus or deep vein thrombosis: guidance from the SSC of the ISTH. J. Thromb. Haemost. 10:698–702. 22332937 10.1111/j.1538-7836.2012.04662.x

[mgg3226-bib-0003] Beckman, M. G. , W. C. Hooper , S. E. Critchley , and T. L. Ortel . 2010 Venous thromboembolism: a public health concern. Am. J. Prev. Med. 38:S495–S501. 20331949 10.1016/j.amepre.2009.12.017

[mgg3226-bib-0004] Borch, K. H. , C. Nyegaard , J.‐B. Hansen , E. B. Mathiesen , I. Njølstad , T. Wilsgaard , et al. 2011 Joint effects of obesity and body height on the risk of venous thromboembolism: the Tromsø Study. Arterioscler. Thromb. Vasc. Biol. 31:1439–1444. 21527750 10.1161/ATVBAHA.110.218925

[mgg3226-bib-0005] Buil, A. , D. Tre , J. C. Souto , M. Germain , M. Rotival , L. Tiret , et al. 2010 C4BPB/C4BPA is a new susceptibility locus for venous thrombosis with unknown protein S–independent mechanism: results from genome‐wide association and gene expression analyses followed by case‐control. Blood 115:4644–4650. 20212171 10.1182/blood-2010-01-263038 PMC2890187

[mgg3226-bib-0006] Chang, C. C. , C. C. Chow , L. C. A. M. Tellier , S. Vattikuti , S. M. Purcell , and J. J. Lee . 2015 Second‐generation PLINK: rising to the challenge of larger and richer datasets. Giga Sci. 4:7. 10.1186/s13742-015-0047-8 PMC4342193 25722852

[mgg3226-bib-0007] Daneshjou, R. , E. R. Gamazon , B. Burkley , L. H. Cavallari , J. A. Johnson , T. E. Klein , et al. 2014 Genetic variant in folate homeostasis is associated with lower warfarin dose in African Americans. Blood 124:2298–2306. 25079360 10.1182/blood-2014-04-568436 PMC4183989

[mgg3226-bib-0008] Dowling, N. F. , H. Austin , A. Dilley , C. Whitsett , B. L. Evatt , and W. C. Hooper . 2003 The epidemiology of venous thromboembolism in Caucasians and African‐Americans: The GATE study. J. Thromb. Haemost. 1:80–87. 12871543 10.1046/j.1538-7836.2003.00031.x

[mgg3226-bib-0009] Duebgen, S. , T. Kauke , C. Marschall , A. Giebl , S. Lison , C. Hart , et al. 2012 Genotype and laboratory and clinical phenotypes of protein s deficiency. Am. J. Clin. Pathol. 137:178–184. 22261441 10.1309/AJCP40UXNBTXGKUX

[mgg3226-bib-0010] Dumitrescu, L. , M. D. Ritchie , K. Brown‐Gentry , J. M. Pulley , M. Basford , J. C. Denny , et al. 2010 Assessing the accuracy of observer‐reported ancestry in a biorepository linked to electronic medical records. Genet. Med. 12:648–650. 20733501 10.1097/GIM.0b013e3181efe2df PMC2952033

[mgg3226-bib-0011] Exome Aggregation Consortium (ExAC) , Cambridge, MA Available at http://exac.broadinstitute.org (accessed June 2016).

[mgg3226-bib-0012] Falush, D. , M. Stephens , and J. K. Pritchard . 2003 Inference of population structure using multilocus genotype data: linked loci and correlated allele frequencies. Genetics 164:1567–1587. 12930761 10.1093/genetics/164.4.1567 PMC1462648

[mgg3226-bib-0013] Fay, M. P. 2010 Confidence intervals that match Fisher's exact or Blaker's exact tests. Biostatistics 11:373–374. 19948745 10.1093/biostatistics/kxp050 PMC2852239

[mgg3226-bib-0014] Feero, W. G . 2004 Genetic thrombophilia. Prim. Care 31:685–709, xi. 15331254 10.1016/j.pop.2004.04.014

[mgg3226-bib-0015] Gandrille, S. , D. Borgel , N. Sala , Y. Espinosa‐Parrilla , R. Simmonds , S. Rezende , et al. 2000 Scientific and Standardization Committee Communication Protein S Deficiency?: A Database of Mutations – Summary of the First Update. Thromb. Haemost. 84:918. 11127877

[mgg3226-bib-0016] Garcia de Frutos, P. , and P. Fuentes‐Prior . 2007 Molecular basis of protein S deficiency. Thromb. Haemost. 98:543–556. 17849042

[mgg3226-bib-0017] Gohil, R. , G. Peck , and P. Sharma . 2009 The genetics of venous thromboembolism. A meta‐analysis involving approximately 120,000 cases and 180,000 controls. Thromb. Haemost. 102:360–370. 19652888 10.1160/TH09-01-0013

[mgg3226-bib-0018] Heeb, M. J. , R. M. Mesters , G. Tans , J. Rosing , and J. H. Griffin . 1993 Binding of protein S to factor Va associated with inhibition of prothrombinase that is independent of activated protein C. J. Biol. Chem. 268:2872–2877. 8428962

[mgg3226-bib-0019] Holzhauer, S. , N. A. Goldenberg , R. Junker , C. Heller , M. Stoll , D. Manner , et al. 2012 Inherited thrombophilia in children with venous thromboembolism and the familial risk of thromboembolism: an observational study. Blood 120:1510–1515. 22581447 10.1182/blood-2012-01-405514 PMC3423787

[mgg3226-bib-0020] ten Kate, M. K. , and J. van der Meer . 2008 Protein S deficiency: a clinical perspective. Haemophilia 14:1222–1228. 18479427 10.1111/j.1365-2516.2008.01775.x

[mgg3226-bib-0021] Kimura, R. , S. Honda , T. Kawasaki , H. Tsuji , S. Madoiwa , S. Yoichi , et al. 2006 Protein S – K196E mutation as a genetic risk factor for deep vein thrombosis. Blood 107:1737–1738. 16461766 10.1182/blood-2005-09-3892

[mgg3226-bib-0022] Kumar, P. , S. Henikoff , and P. C. Ng . 2009 Predicting the effects of coding non‐synonymous variants on protein function using the SIFT algorithm. Nat. Protoc. 4:1073–1081. 19561590 10.1038/nprot.2009.86

[mgg3226-bib-0023] Lek, M. , K. Karczewski , E. Minikel , K. Samocha , E. Banks , T. Fennell , et al. 2015 Analysis of protein‐coding genetic variation in 60,706 humans. bioRxiv 1–26. 10.1038/nature19057 PMC5018207 27535533

[mgg3226-bib-0024] Li, B. , D. J. Liu , and S. M. Leal . 2013 Identifying rare variants associated with complex traits via sequencing. Curr. Protoc. Hum. Genet. 78:1–22. 10.1002/0471142905.hg0126s78 PMC3830981 23853079

[mgg3226-bib-0025] Makris, M. , M. Leach , N. J. Beauchamp , M. E. Daly , P. C. Cooper , K. Kingsley , et al. 2000 Genetic analysis, phenotypic diagnosis, and risk of venous thrombosis in families with inherited deficiencies of protein S Genetic analysis, phenotypic diagnosis, and risk of venous thrombosis in families with inherited deficiencies of protein S. Blood 95:1935–1941. 10706858

[mgg3226-bib-0026] Martinelli, I. , T. Battaglioli , P. Bucciarelli , S. M. Passamonti , and P. M. Mannucci . 2004 Risk factors and recurrence rate of primary deep vein thrombosis of the upper extremities. Circulation 110:566–570. 15262837 10.1161/01.CIR.0000137123.55051.9B

[mgg3226-bib-0027] Middeldorp, S. , and A. van Hylckama Vlieg . 2008 Does thrombophilia testing help in the clinical management of patients? Br. J. Haematol. 143:321–335. 18710381 10.1111/j.1365-2141.2008.07339.x

[mgg3226-bib-0028] Mustafa, S. , I. Pabinger , and C. Mannhalter . 1995 Protein S deficiency type I: identification of point mutations in 9 of 10 families. Blood 86:3444–3451. 7579449

[mgg3226-bib-0029] NHLBI GO Exome Sequencing Project (ESP) . Exome Variant Server. Available at http://evs.gs.washington.edu/EVS/. (accessed June 2014).

[mgg3226-bib-0030] Perera, M. A. , L. H. Cavallari , N. A. Limdi , E. R. Gamazon , A. Konkashbaev , R. Daneshjou , et al. 2013 Genetic variants associated with warfarin dose in African‐American individuals: a genome‐wide association study. Lancet 6736:1–7. 10.1016/S0140-6736(13)60681-9 PMC3759580 23755828

[mgg3226-bib-0031] Purcell S., B. Neale , K. Todd‐Brown , L. Thomas , M. A. R. Ferreira , D. Bender , J. Maller , et al. 2007 PLINK: a tool set for whole‐genome association and population‐based linkage analyses. Am. J. Hum. Genet. 81:559–575. 17701901 10.1086/519795 PMC1950838

[mgg3226-bib-0032] Raskob, G. E. , R. Silverstein , D. W. Bratzler , J. A. Heit , and R. H. White . 2010 Surveillance for deep vein thrombosis and pulmonary embolism: recommendations from a national workshop. Am. J. Prev. Med. 38:S502–S509. 20331950 10.1016/j.amepre.2010.01.010

[mgg3226-bib-0033] Ritchie, M. D. , J. C. Denny , D. C. Crawford , A. H. Ramirez , J. B. Weiner , J. M. Pulley , et al. 2010 Robust replication of genotype‐phenotype associations across multiple diseases in an electronic medical record. Am. J. Hum. Genet. 86:560–572. 20362271 10.1016/j.ajhg.2010.03.003 PMC2850440

[mgg3226-bib-0034] Robbins, C. , J. B. Torres , S. Hooker , C. Bonilla , W. Hernandez , A. Candreva , et al. 2007 Confirmation study of prostate cancer risk variants at 8q24 in African Americans identifies a novel risk locus. Genome Res. 17:1717–1722. 17978284 10.1101/gr.6782707 PMC2099580

[mgg3226-bib-0035] Roberts, L. N. , R. K. Patel , and R. Arya . 2009 Venous thromboembolism and ethnicity. Br. J. Haematol. 146:369–383. 19552721 10.1111/j.1365-2141.2009.07786.x

[mgg3226-bib-0036] Roden, D. M. , J. M. Pulley , M. A. Basford , G. R. Bernard , E. W. Clayton , J. R. Balser , et al. 2008 Development of a large‐scale de‐identified DNA biobank to enable personalized medicine. Clin. Pharmacol. Ther. 84:362–369. 18500243 10.1038/clpt.2008.89 PMC3763939

[mgg3226-bib-0037] Rosenberg, N. A. , L. Huang , E. M. Jewett , Z. A. Szpiech , I. Jankovic , and M. Boehnke . 2010 Genome‐wide association studies in diverse populations. Nat. Rev. Genet. 11:356–366. 20395969 10.1038/nrg2760 PMC3079573

[mgg3226-bib-0038] Rosendaal, F. R. , and P. H. Reitsma . 2009 Genetics of venous thrombosis. J. Thromb. Haemost. 7(Suppl. 1):301–304. 19630821 10.1111/j.1538-7836.2009.03394.x

[mgg3226-bib-0039] van Wijnen, M. , J. Stam , G. Chang , J. Meijers , P. Reitsma , R. Bertina , et al. 1998 Characterization of mini‐protein S, a recombinant variant of protein S that lacks the sex hormone binding globulin‐like domain. Biochem. J. 330:389–396. 9461535 10.1042/bj3300389 PMC1219152

[mgg3226-bib-0040] Zakai, N. A. , and L. A. McClure . 2011 Racial differences in venous thromboembolism. J. Thromb. Haemost. 9:1877–1882. 21797965 10.1111/j.1538-7836.2011.04443.x

