# Supplementary material for: Population‐specific single‐nucleotide polymorphism confers increased risk of venous thromboembolism in African Americans
Source: Mol Genet Genomic Med. 2016 Jun 21;4(5):513–20. doi: 10.1002/mgg3.226 (PMC5023936; doi:10.1002/mgg3.226)
Supplement: Supplementary file 1 — Figure S1. Analysis process for identifying variants of interest in an African American family with a history of venous thromboembolism. Figure S2. Description of Primers used for Pyrosequencing of SNP. Figure S3. Description of Primers used for Taqman SNP genotyping assay. Table S1. Clinical data of the African American family with hereditary VTE. Table S2. Covariates for Warfarin and Vanderbilt subcohorts. *Denotes statistical difference between cases and controls in a subcohort. Table S3. Logistic model for population cohort using height, weight, and risk variant. 98 samples were excluded due to missingness in height or weight. Exhibit S1. Quality Control Measures. [file MGG3-4-513-s001.docx]

**Figure S1:** Analysis process for identifying variants of interest in an African American family with a history of venous thromboembolism.


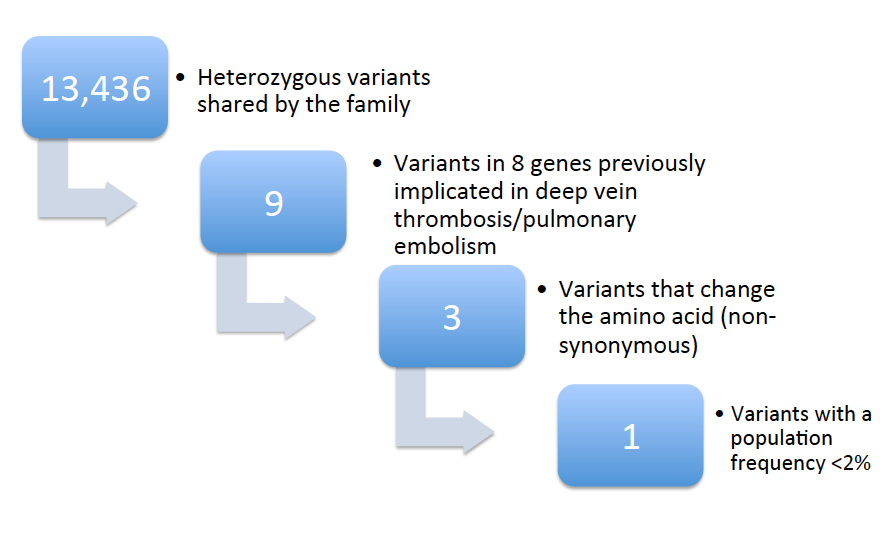


**Figure S2:** Description of Primers used for Pyrosequencing of SNP

| **SNP** | **Method** | **Primer (all 5’ to 3’)** | | |
| --- | --- | --- | --- | --- |
|  |  | **PCR Forward** | **PCR Reverse** | **Sequencing** |
| rs138925964 | Pyro-sequencing | Biotin- CGTGGATGGACGAATATTCAAGG | GTGCTGAGGGTTGGCATGTAA | GGGTTGGCATGTAAAT |

**Figure S3:** Description of Primers used for Taqman SNP genotyping assay

| **SNP** | **Method** | **Forward Primer Sequence**  **(5’ to 3’)** | **MGB probe Sequences 1 and 2 (5’ to 3’)** | **Reverse Primer Sequence (5’ to 3’)** |
| --- | --- | --- | --- | --- |
| rs138925964 | Taqman SNP genotyping assay | CCCGTGGATGGACGAATATTCA | TTGGCATGTAAATGTGACCT  TTGGCATGTAAATATGACCT | TTTTAAATAGATAATGTATCCAGTGCTGAGGG |

**Table S1:** Clinical data of the African American family with hereditary VTE.

| Subject Number | 3 | 2 | 1 |
| --- | --- | --- | --- |
| Past Medical History | 4-5 DVTs in both extremities, 2 PEs, cerebral vascular accident, seizure disorder, hypertension, and Protein S deficiency | 5 left extremity DVTs, hypertension, thrombotic thrombocytopenic purpura, Protein S deficiency, Systemic Lupus Erythematosus, multiple nonviable pregnancies | Right Lower Extremity DVT, Diverticulitis |
| Social Factors | 11.5 pack-years of cigarettes quit 30 years prior, denies EtOH | Drug abuse and tobacco use | Tobacco use |
| Remarkable Labs | >5 years after first DVT; 11 days off warfarin:  high thrombin time, high fibrinogen, high plasminogen, high total Protein S (142%), low Free Protein S (55%) | 1 year after 1^st^ DVT, not on warfarin:  low to normal total protein S (68%), low free protein S (20%), Anti-SSA antibodies (interpreted as Sjogren’s syndrome), High Anti-B2 glycoprotein IGA | Work up 1:  (After DVT, on heparin before warfarin.)  Low Free Protein S (53%), High Factor V  Work up 2: (8 months after 1^st^ workup, on low molecular weight heparin while warfarin held for procedure)  Low free protein S (44%), High thrombin time |
| Unremarkable Labs | Normal thrombin, d-dimer, antithrombin, Protein C function, Lupus inhibitor, and Dilute Russells Viper Venom | Normal antithrombin, protein C, d-dimer, ACA IGM, ACA IGA, ACA IGG, Anti-B2 glycoprotein IGG, IGM | Work up 1:  Normal anthithrombin, Protein C function, Total Protein S, Lupus inhibitor, Diulte RVV, Factor II, ACL antibodies.  Work up 2:  Normal antithrombin, Plasminogen, Protein C function, total Protein S, Lupus inhibitor, Dilute RVV, Thrombin, d-dimer, fibrinogen |
| Modifying genetics | Sickle cell anemia carrier | None | None |

**Table S2:** Covariates for Warfarin and Vanderbilt sub-cohorts. * denotes statistical difference between cases and controls in a sub-cohort.

| Covariate | Warfarin Cases | Warfarin Controls | Vanderbilt BioVU Cases | Vanderbilt BioVU Controls |
| --- | --- | --- | --- | --- |
| Mean Age at enrollment | 54.47* | 58.54* | 32.90* | 77.36* |
| Sex (%F) | 72.3% | 67.2% | 65.4% | 58.9% |
| Mean weight (kg) | 97.50* | 88.26* | 84.00* | 81.95* |
| Mean height (cm) | 167.59 | 168.07 | 158.93* | 169.08* |

**Table S3:** Logistic model for population cohort using height, weight, and risk variant. 98 samples were excluded due to missingness in height or weight.

| Variable | coefficient | p-value |
| --- | --- | --- |
| Height | -0.036 | 0.035 |
| Weight | 0.019 | 1.01E-6 |
| *PROS1* V510M | 1.411 | 2.81E-7 |

**Exhibit S1:** Quality Control Measures

**Warfarin Sub-cohort**

The warfarin sub-cohort consisted of genotyped samples and exome-sequenced samples.

A full description of QC measures for the exome-sequenced cohort can be found in the Daneshjou et al., Blood 2014 paper. Briefly, the exomes were sequenced at 30x coverage, and concordance with a subset of the exomes that had undergone genotyping was 99.8%. Relatedness in the exome sequencing set was assessed using identity by state analysis, as previously described in the Daneshjou et al, Blood 2014 paper.

The *PROS1* V510M genotyped samples came from the University of Florida and the University of Illinois at Chicago; however, the DNA was genotyped at the same site. 79 out of 205 genotyped samples had previously been run on a genomwide SNP chip for genomewide association studies and were confirmed to be unrelated (Klein et al. 2009; Perera et al. 2013). 2 of the variant carriers were among the genotyped samples, 4 were not. However, there was no difference in the risk allele distribution in the cases or controls between the exome sequenced data (all confirmed to be unrelated) and the genotyped samples (see below under “Comparing allele frequencies between sub-cohorts”).

**Vanderbilt BioVU Sub-cohort**

The custom Taqman SNP Genotyping assay was validated on 5 homozygous reference and 5 heterozygous samples from the warfarin genotyped sub-cohort. 78 of the samples had genomewide SNP chip data available, which was used to test for relatedness through identity by state analysis; none of these samples were related. Additionally, the risk allele distribution between cases in this cohort and cases in the other two cohorts were the same (see below).

**Comparing allele frequencies between sub-cohorts**

Additionally, we compared the distribution of the variant among the cases and controls between the differently processed cohorts: the exome sequenced warfarin sub-cohort, the genotyped warfarin sub-cohort, and the genotyped Vanderbilt BioVU sub-cohort. Comparisons were done using the Fisher’s Exact Test. All statistical analysis was done in the statistical programming package R (v. 2.15.3); Fisher’s Exact Test was done using the exactci_1.2-0 library in R.(Fay 2010).

There was no difference in the risk allele distribution between the cases in the Vanderbilt BioVU sub-cohort compared to the warfarin exome cohort (p> 0.1), the Vanderbilt BioVU sub-cohort compared to the warfarin genotyped cohort (p> 0.1), the warfarin exome sub-cohort compared to the warfarin genotyped sub-cohort (p >0.1). There was also no difference in the risk allele distribution between the controls in the Vanderbilt BioVU sub-cohort compared to the warfarin exome sub-cohort (p>0.1), the Vanderbilt BioVU sub-cohort compared to the warfarin genotyped sub-cohort (p>0.1), or the warfarin exome sub-cohort compared to the warfarin genotyped sub-cohort (p>0.1). This makes it unlikely that our finding was driven by a single sub-cohort.
